# Effects of decellularized extracellular matrix derived from Jagged1-treated human dental pulp stem cells on biological responses of stem cells isolated from apical papilla

**DOI:** 10.3389/fcell.2022.948812

**Published:** 2022-08-23

**Authors:** Suphalak Phothichailert, Nunthawan Nowwarote, Benjamin P.J. Fournier, Vorapat Trachoo, Sittiruk Roytrakul, Worachat Namangkalakul, Thanaphum Osathanon

**Affiliations:** ^1^ Dental Stem Cell Biology Research Unit, Faculty of Dentistry, Chulalongkorn University, Bangkok, Thailand; ^2^ Universite Paris Cite, Faculty of Dentistry, Department of Oral Biology, Paris, France; ^3^ Centre de Recherche des Cordeliers, Sorbonne Universite, INSERM UMRS, Molecular Oral Pathophysiology, Paris, France; ^4^ Department of Oral and Maxillofacial Surgery, Faculty of Dentistry, Chulalongkorn University, Bangkok, Thailand; ^5^ Proteomics Research Laboratory, Genome Institute, National Center of Genetic Engineering and Biotechnology, National Science and Technology Development Agency, Pathumthani, Thailand; ^6^ Department of Anatomy, Faculty of Dentistry, Chulalongkorn University, Bangkok, Thailand

**Keywords:** biocompatibility, decellularized, differentiation, Jagged1, stem cells isolated from apical papilla

## Abstract

**Objective:** Indirect Jagged1 immobilization efficiently activates canonical Notch signaling in human dental pulp stem cells (hDPSCs). This study aimed to investigate the characteristics of the Jagged1-treated hDPSC-derived decellularized extracellular matrix (dECM) and its biological activity on odonto/osteogenic differentiation of stem cells isolated from apical papilla (SCAPs).

**Methods:** Bioinformatic database of Jagged1-treated hDPSCs was analyzed using NetworkAnalyst. hDPSCs seeded on the Jagged1 immobilized surface were maintained with normal or osteogenic induction medium (OM) followed by decellularization procedure, dECM-N, or dECM-OM, respectively. SCAPs were reseeded on each dECM with either the normal medium or the OM. Cell viability was determined by MTT assay. Characteristics of dECMs and SCAPs were evaluated by SEM, EDX, immunofluorescent staining, and alcian blue staining. Mineralization was assessed by alizarin red S, Von Kossa, and alkaline phosphatase staining. Statistical significance was considered at *p* < 0.05.

**Results:** RNA-seq database revealed upregulation of several genes involved in ECM organization, ECM–receptor interaction, and focal adhesion in Jagged1-treated hDPSCs. Immobilized Jagged1 increased the osteogenesis of the hDPSC culture with OM. dECMs showed fibrillar-like network structure and maintained major ECM proteins, fibronectin, type I-collagen, and glycosaminoglycans. A decrease in calcium and phosphate components was observed in dECMs after the decellularized process. Cell viability on dECMs did not alter by 7 days. Cell attachment and f-actin cytoskeletal organization of SCAPs proliferated on Jagged1-treated dECMs were comparable to those of the control dECMs. SCAPs exhibited significantly higher mineralization on dECM-N in OM and markedly enhanced on dECM-OM with normal medium or OM conditions.

**Conclusion:** Jagged1-treated hDPSC-derived dECMs are biocompatible and increase odonto/osteogenic differentiation of SCAPs. The results suggested the potential of Jagged1 dECMs, which could be further developed into ECM scaffolds for application in regenerative medicine.

## Introduction

Notch is a crucial signaling pathway that regulates embryonic bone formation as well as adult bone healing and regeneration. Indeed, canonical Notch signaling controls cell proliferation, differentiation, and self-renewal in several cell types, such as the hematopoietic stem cell, neural stem cells, and skeletal muscle cells (Mancini et al., 2005; Mourikis et al., 2012; Luo et al., 2019). Jagged1 is one of the canonical Notch ligands that activates this signaling cascade in mammalian cells (Tien, Rajan, and Bellen 2009; Dishowitz et al., 2014). The binding of ligands and receptors results in the activation of the Notch intracellular domain (NICD) using the multi-stage proteolytic processes by ADAM (a disintegrin and metalloprotease) protease and *γ*-secretase complex (Youngstrom et al., 2016; Zhang et al., 2016). NICD, which later translocates to the nucleus, interacts with CSL transcription factors and leads to initiating the expression of target genes such as HES/HEY (hairy and enhancer of split/hairy and enhancer of split related with YRPW motif) (Zhang et al., 2016; Luo et al., 2019).

Extracellular matrix (ECM) is currently considered as the biomaterial of choice for tissue regeneration (Zhong et al., 2019) due to its ability to control cell behavior, adhesion, and functions (Khalili and Ahmad 2015). Decellularized ECM scaffolds can be prepared using a variety of chemical and mechanical techniques (Faulk et al., 2015; Gilpin and Yang 2017). Bone ECM stimulates the formation of new bone from mesenchymal stem cells, osteoblasts, and osteocytes (Lin et al., 2020). Bone marrow mesenchymal stem cell (MSC)-derived ECM encourages cell adhesion and osteogenic marker gene expression (Chi et al., 2020). Interestingly, previous reports revealed the favorable *in vitro* effects of ECM scaffolds derived from dental and oral cells. Human dental pulp stem cell (hDPSC)-derived ECM scaffold enhanced odonto/osteogenic differentiation of hDPSCs, human periodontal ligament stem cells (hPDLSCs), and human MSCs (Ravindran et al., 2014a), and supported neovascularization by upregulation of pro-angiogenic growth factors (Ravindran et al., 2014b). Furthermore, ECM scaffolds are engaged and used in various fields of regenerative medicine (Ott et al., 2008; Gilpin and Yang 2017; Scarritt et al., 2019). These suggest the suitable functions of ECM for utilization in mineralized tissue engineering.

Immobilized Jagged1-treated human dental pulp cells (hDPCs) exhibited the upregulation of genes that are involved in ECM components as well as ECM-receptor interaction and focal adhesion-related pathways, such as ECM organization, collagen biosynthesis, and modification of enzymes (Manokawinchoke et al., 2017). In addition, Jagged1 plays a pivotal role in the cell-matrix interaction (Nehring et al., 2005). Therefore, indirect immobilized Jagged1 could modulate the biological properties of hDPSC-derived ECM through canonical Notch signaling stimulation, which suggested the benefits of ECM for prospective applications in dental tissue regeneration.

Collectively, ECM-derived from dental stem cells has the potential for enabling and enhancing odonto/osteogenic differentiation of MSCs. However, the effects of ECM scaffolds on stem cells isolated from apical papilla (SCAPs), another type of dental stem cell found in the apical tissues of the immature tooth root, have not been extensively studied. The previous report demonstrated that the properties of SCAPs are important for developing regenerative endodontic therapies and achieving revascularization of the root canal system (Raddall et al., 2019). These cells are the source of odontoblast-like cells that are responsible for the root dentin formation and have the potential for dentin engineering (Huang et al., 2008). In this study, we investigated the characteristics of decellularized ECM derived from immobilized Jagged1-treated hDPSCs and their biological activity on SCAPs, *in vitro*.

## Materials and methods

### Cell isolation and culture

The experimental protocols were approved by the Human Research Ethics Committee, Faculty of Density, Chulalongkorn University (approval no. 106/2021). Third molars scheduled for surgical removal according to the patient’s treatment plan were obtained for cell isolation. Informed consent was performed. In brief, dental pulp and apical papilla tissues were collected and minced. Cell explantation was performed to isolate cells from both tissues. Cells were cultured in Dulbecco’s modified Eagle’s medium (DMEM) (Gibco, United States) supplemented with 10% fetal bovine serum (Gibco, United States), 2 mM l-glutamine, and 100 units/ml penicillin, 100 μg/ml streptomycin, and 250 ng/ml amphotericin B (Gibco, United States) (growth medium). Cells were incubated in a humidified atmosphere with 5% CO_2_ at 37°C, and the culture medium was changed every 2 days. Cells were then sub-cultured, and all experiments used the cells from passages 3 to 5.

### Flow cytometry

To identify the characteristics of mesenchymal stem cells, surface marker protein expression was evaluated by flow cytometry. hDPSCs and SCAPs were stained with FITC-conjugated CD44 (BD Bioscience Pharmingen, United States), FITC-conjugated CD73 (BD Bioscience Pharmingen, United States), PE-conjugated CD105 (Immuno Tools, Germany), APC-conjugated CD90 (Immuno Tools, Germany), and PerCP-conjugated CD45 antibodies (Immuno Tools, Germany) (1:50 dilution for all antibodies). Mouse IgG isotype was used as the control. The stained cells were analyzed by FACS^Calibur^ Flow Cytometer (Becton Dickinson, Worldwide Inc, United States).

### Osteogenic differentiation

To induce osteogenic differentiation, 5×10^4^ cells were seeded into a 24-well plate and cultured with a growth medium until confluence. Cells were maintained with an osteogenic induction medium (OM) which contained a growth medium supplemented with 50 μg/ml ascorbic acid (Sigma-Aldrich, United States), 5 mM beta-glycerophosphate (Sigma-Aldrich, United States), and 250 nM dexamethasone (Sigma-Aldrich, United States) for 14 days.

For mineralization assay, cells were fixed with 4% formaldehyde in phosphate buffer saline (PBS) for 5 min and washed with deionized water. The samples were then stained with Alizarin Red S (ARS) solution (pH 4.1) (Sigma-Aldrich, United States) for 5 min at room temperature (RT) and washed with deionized water. Stained mineral deposits were observed under a microscope and further solubilized with 10% cetylpyridium chloride monohydrate in 10 mM sodium phosphate solution for 20 min. The optical density was measured at 570 nm with a microplate reader (ELx800, BioTek, United States). For Von Kossa staining, cells were fixed with 4% formaldehyde in PBS and stained with 5% silver nitrate in sterile deionized water under UV light for 5 min at RT.

For alkaline phosphatase assay, cells were fixed with 4% formaldehyde in PBS and washed with deionized water. Subsequently, cells were stained with nitro blue tetrazolium chloride and 5-bromo-4-chloro-3-indolyl phosphate tablets (Roche, United States) in sterile deionized water for 30 min in the dark condition.

### Adipogenic differentiation

Cells were seeded at a density of 1.25×10^4^ cells/well and maintained with an adipogenic induction medium, which consisted of a growth medium supplemented with 1 mM 3-isobutyl-1-methylxanthine (IBMX, Thermo Fisher Scientific, United States), 0.1 mg/ml insulin (Sigma-Aldrich, United States), 1 μM dexamethasone, and 0.2 mM indomethacin (Sigma-Aldrich, United States) for 16 days. The culture medium was refreshed every 3 days. The intracellular lipid accumulation was examined using Oil Red O staining. Cells were fixed in 4% formaldehyde in PBS for 15 min, rinsed with deionized water, and stained with Oil Red O solution (Sigma-Aldrich, United States) for 15 min at RT.

### High-throughput RNA sequencing data analysis

RNA sequencing data of hDPCs seeded on indirect immobilized Jagged1 surface for 24 h were downloaded from the NCBI Sequence Read Archive and NCBI Gene Expression Omnibus (SRP100068 and GSE94989, respectively). The gene related to ECM organization was identified and represented by the heat map. The raw expression was analyzed using NetworkAnalyst (Xia et al., 2015; Zhou et al., 2019). The RNA was identified and an expression heatmap was generated using Heatmapper (Babicki et al., 2016).

### Jagged1 immobilization

Recombinant human (rh) Jagged1/FC protein (R&D Systems, United States) was prepared for coating the surface of the tissue culture plate according to the previous report (Manokawinchoke et al., 2017). In short, tissue culture plates were incubated with 50 μg/ml recombinant protein G for 16 h and washed with sterile PBS. Next, the surfaces were incubated with 10 mg/ml bovine serum albumin for 2 h, washed with PBS, and then incubated with 10 nM rhJagged1/FC for 2 h. The human IgG Fc fragment (hFc) (Jackson ImmunoResearch Laboratory, United States) was used as the immobilization control.

### Generation of decellularized extracellular matrix

Cells were seeded on rhJagged1/Fc protein-coated 24-well plates at 5 × 10^5^ cells and maintained with a growth medium in a humidified atmosphere with 5% CO_2_ at 37°C. To generate Jagged1-treated hDPSC-derived ECM, the culture medium was changed to a normal medium (N medium), which is a growth medium supplemented with 50 μg/ml ascorbic acid, or an OM on day 7. The culture medium was changed every 2 days. Cells were then harvested on day 21.

For the preparation of Jagged1-treated hDPSC-derived decellularized ECM (dECM), samples were incubated with 0.5% Triton X-100 in 20 mM ammonium hydroxide for 5–10 min for removing all DNA components and washed with a protease inhibitor in PBS. Then, 0.0025% deoxyribonuclease in sterile PBS was added and incubated for 5–15 min at RT to break down DNA fragments and washed with a protease inhibitor in PBS. The hDPSC-derived dECM was preserved by avoiding the dry atmosphere or kept in deionized water at 4°C.

### Scanning electron microscopy and energy-dispersive X-ray spectrometry

The specimens were fixed with 3% glutaraldehyde in PBS for 30 min. The samples were dehydrated with serial graded ethanol (30–100%) and hexamethyldisiloxane was added for 5 min, dried, and the gold coating was performed. The cells and dECM morphology were observed using SEM (Quanta 250, FEI, United States). For element component detection, specimens without gold coating were examined using EDX (JSM-5410LV, JEOL, Japan).

### Characterization of SCAPs on dECM

SCAPs were seeded at a density of 2.5 × 10^4^ cells on hDPSC-derived dECMs and incubated with a growth medium at 37 °C in a humidified atmosphere with 5% CO_2_ for 30 min, 24 h, and 7 days. Immunofluorescence, SEM, and EDX were performed to observe cell morphology, cell attachment, and spreading, respectively.

### Cell viability assay

Cell viability was measured using the 3-(4,5-dimethylthiazol-2-yl)-2,5-diphenyltetrazolium bromide (MTT) assay (Tocris Bioscience, UK). SCAPs were seeded on hDPSC-derived dECM. On days1, 3, and 7, MTT solution (0.5 mg/ml) was added and incubated at 37 °C for 15 min. The formazan crystals were dissolved in dimethylsulfoxide and glycine buffer. The absorbance at 570 nm was measured using a microplate reader (Molecular Devices, United States).

### Immunofluorescence staining

hDPSC-derived dECMs were assessed for fibronectin and type I-collgen expression. The morphological appearance of the SCAPs seeded on hDPSC-derived dECMs was also evaluated by the F-actin organization using phalloidin staining (1:1000, Invitrogen, United States). Briefly, dECM or cells were fixed in 4% formaldehyde in PBS and permeabilized with 0.1% TritonX-100. Non-specific blocking was performed by incubating with 10% fetal bovine serum at 4 °C overnight. The cells were stained with primary antibodies against type I collagen (1:200, Abcam, UK) or fibronectin (1:500, Invitrogen, United States) for 2 h, and further incubated with secondary antibodies. The samples were then incubated with biotinylated anti-rabbit IgG antibodies (Sigma-Aldrich, United States) at a dilution of 1:2000 for 40 min and Strep-Rhodamine (Invitrogen, United States) was stained at 1:500. DAPI (Invitrogen, United States) was used for nuclear counterstaining. Visualization was performed using the fluorescence microscope with a ApoTome system (Carl Zeiss, Germany).

### Glycosaminoglycans staining

dECM were fixed with 0.1% glutaraldehyde (Sigma-Aldrich, United States) in PBS for 20 min. dECMs were stained with 1% w/v Alcian Blue solution (Sigma-Aldrich, United States) in 0.1 M HCl, incubated at RT for 24 h, and rinsed with 0.1 M HCl and PBS. Stained dECM was observed by microscope.

### Statistical analysis

All experiments were performed using cells from four different donors. The Kruskal–Wallis test followed by a pairwise comparison was employed for more than three group comparisons. The Mann–Whitney *U* test was used for statistical analysis of two group comparisons (GraphPad Software, United States). Statistical significance was defined at *p* < 0.05.

## Results

### Characterization of hDPSCs

**FIGURE 1:**
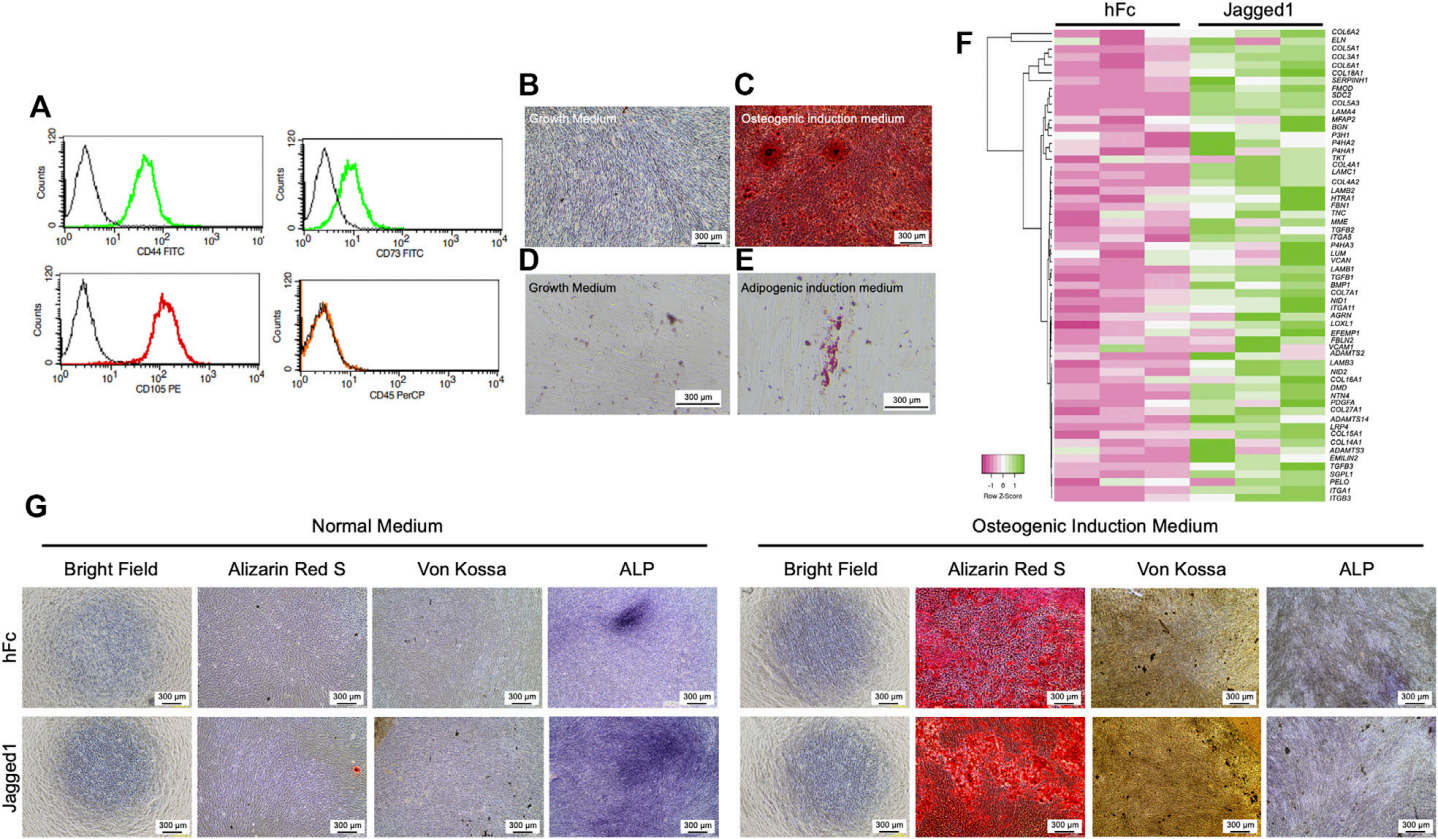
Jagged1 promotes mineralization and ECM gene expression in hDPSCs. Human dental pulp stem cells (hDPSCs) were characterized by flow cytometry to examine surface protein marker expression **(A)**. Mineralization was examined using Alizarin Red S staining on day 14 after osteogenic induction **(B–C)**. Intracellular lipid accumulation was detected using Oil Red O staining on day 16 after adipogenic induction **(D–E)**. The effect of Jagged1 on hDPSCs was examined. Bioinformatic analysis of RNA sequencing data of genes related to ECM organization was illustrated by heatmap **(F)**. Morphology, mineralization, and alkaline phosphatase enzymatic activity (ALP) were examined **(G)**.

The characteristics of the human dental pulp stem cells (hDPSCs) used in this study were investigated with cell surface marker expression by flow cytometry. hDPSCs were positive for mesenchymal stem cell surface markers, CD44, CD73, and CD105 but negative for a hematopoietic surface marker, CD45 (Figure 1A). Osteogenic and adipogenic differentiation potentials of hDPSCs were also examined. The mineral deposition was markedly detected by Alizarin Red S (ARS) staining after maintaining those cells with osteogenic induction medium (OM) for 14 days (Figures 1B,C). When hDPSCs were cultured with an adipogenic induction medium, the intracellular lipid droplets were observed with Oil Red O staining on day16 (Figures 1D,E). These results confirmed that isolated hDPSCs were mesenchymal stem cell populations.

### Jagged1-treated hDPSCs modulated genes related to ECM organization category

hDPSCs cultured on recombinant human Jagged1-treated surfaces (Jagged1-treated hDPSCs) significantly increased the expression of several extracellular matrix (ECM) organization genes according to the bioinformatic analysis of the RNA sequencing database (SRP100068 and GSE94989). The upregulated ECM component genes in Jagged1-treated hDPSCs, including laminin subunits (*LAMA4, LAMB1, LAMB2,* and *LAMC1*), elastins (*VCAN, ELN,* and *EMILIN2*), proteoglycans (*FMOD* and *LUM*), glycoproteins (*FBN1, NID1, NID2,* and *EFEMP1*), fibronectin (*TNC*), integrins (*ITG, ITGA5,* and *ITGA11*), and glycosaminoglycans (*AGRN*) as shown in the heat map (Figure 1F). Moreover, several collagen genes were upregulated in the Jagged1-treated hDPSCs compared with the hFC-treated control such as *COL5A1, COL3A1, COL4A1, COL27A1,* and *COL5A3*. The expression profile also demonstrated an increase in the transcription level of collagen synthesis and assembly-associated genes in Jagged1-treated hDPSCs such as *P4HA3, P4HA1, ADAMTS14, ADAMTS3*, and *LOXL1* (Figure 1F).

### Jagged1 promoted mineralization in hDPSCs

Next, the osteogenic differentiation potential of hDPSCs on indirect Jagged1-treated tissue culture surfaces was assessed after maintaining those cells in either a growth medium supplemented with 50 μg/ml ascorbic acid (a normal medium) or OM for 21 days. In a normal medium condition, the mineral deposition of hDPSCs was not observed in both Jagged1-and hFc-treated surfaces, while the ALP staining was positive (Figure 1G). ALP activity was higher in Jagged1-treated hDPSCs compared with the hFc control. When maintaining hDPSCs on Jagged1-treated surfaces with OM, significant enhancing mineralization was observed compared to those cells maintained with a normal medium. Jagged1 immobilization also more robustly increased the mineral deposits of hDPSCs than those of the hFc-treated control in an OM condition (Figure 1G). Therefore, Jagged1 immobilized surfaces effectively enhanced an *in vitro* osteogenic differentiation of hDPSCs.

### Characteristics and morphological appearance of decellularized ECM derived from Jagged1-treated hDPSCs

**FIGURE 2:**
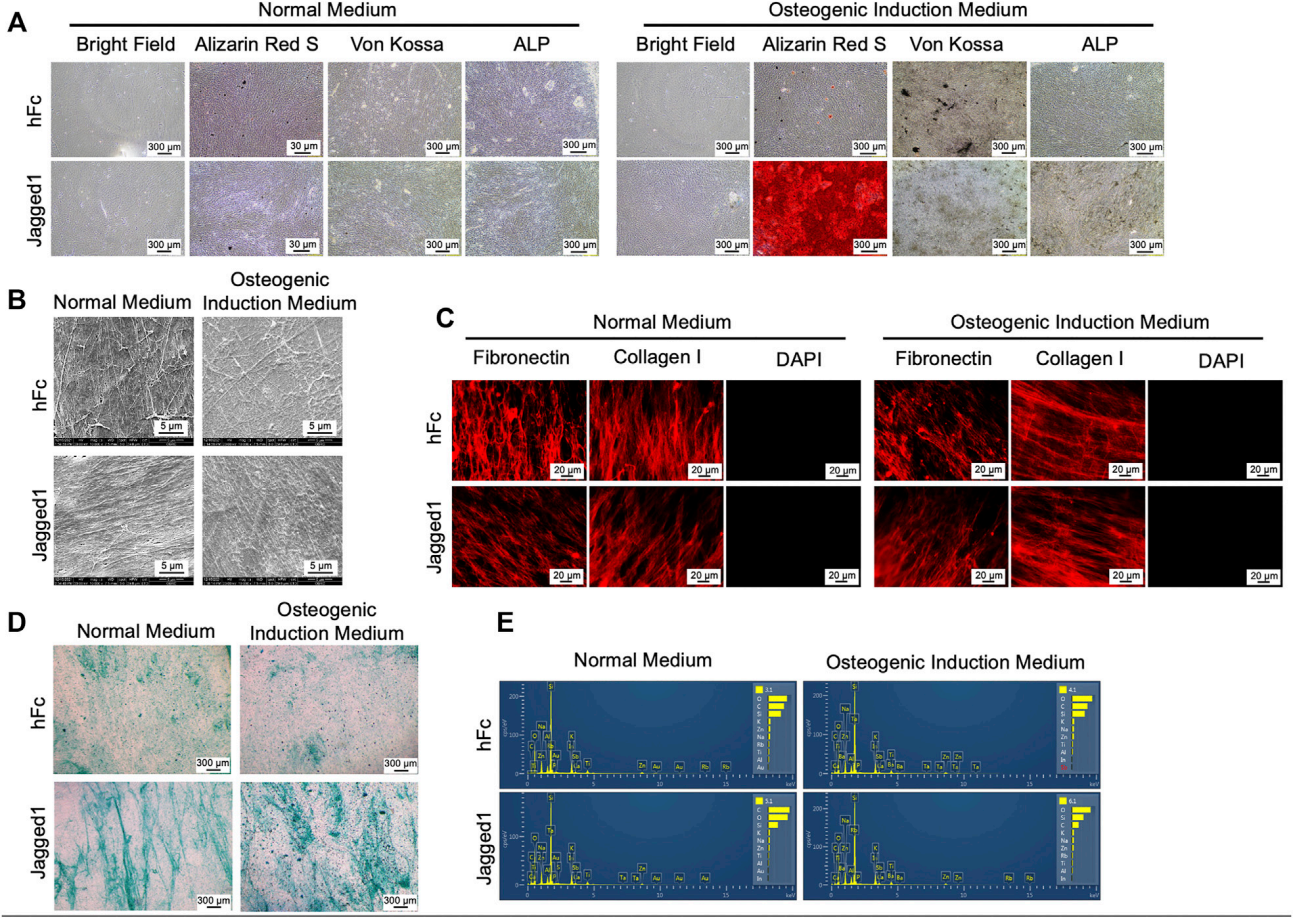
Characterization of decellularized extracellular matrix (dECM). Morphology, mineralization, and alkaline phosphatase enzymatic activity (ALP) were examined **(A)**. Ultrastructure of dECM was observed using scanning electron microscopic analysis **(B)**. Fibronectin and type I-collagen were determined using immunofluorescence staining **(C)**. The genetic component was stained using DAPI **(C)**. Glycosaminoglycans deposition was detected by Alcian Blue staining **(D)** Chemical composition of dECM was examined using energy-dispersive X-ray spectrometry **(E)**. dECM-N; decellularized extracellular matrix derived from maintaining cells in normal medium, dECM-OM; decellularized extracellular matrix derived from maintaining cells in osteogenic medium.

After culturing hDPSCs on Jagged1-or hFc-treated surfaces with the normal medium or OM for 21 days, we investigated the characteristics of ECM after conducting the decellularization process. The bright-field microscopic observation indicated the absence of cells. The dECM derived from the normal medium condition, both Jagged1 and hFc dECM-N, showed negative for ARS, Von Kossa, and ALP staining. In contrast, when maintaining the hDPSC culture in the osteogenic induction conditions (dECM-OM), the calcium deposition remained in Jagged1-treated dECM-OM compared to that of hFc-treated dECM-OM control after eliminating cellular components (Figure 2A). Scanning electron microscopy (SEM) analysis of Jagged1-treated hDPSC-derived dECMs revealed the massive well-arranged fibrillar ECM networks in both the normal medium and OM conditions (Figure 2B). ECM proteins were visualized after decellularization by immunofluorescence staining. We found that fibronectin and type I-collagen were preserved in dECM derived from Jagged1-and hFc-treated surfaces. Moreover, the absence of DAPI nuclei staining in dECM-N and dECM-OM indicated that the decellularizing procedure was effective in removing genetic materials (Figure 2C). Proteoglycans accumulation in dECM was also observed by Alcian Blue staining after decellularization in order to detect glycosaminoglycans (GAGs). The result showed that GAGs deposition was increased in Jagged1-treated dECMs in both the normal medium and OM conditions when compared with the hFc control (Figure 2D). We used energy dispersive X-ray spectrometry (EDX) to investigate the chemical elements of dECM. The types of chemical components exhibited on surfaces were similar in both dECM derived from Jagged1-and hFc-treated hDPSCs with the normal medium and OM conditions (Figure 2E). The findings confirmed that dECMs were deprived of cellular components but retained the main ECM proteins.

### Biological responses of SCAPs on dECM derived from Jagged1-treated hDPSCs

**FIGURE 3:**
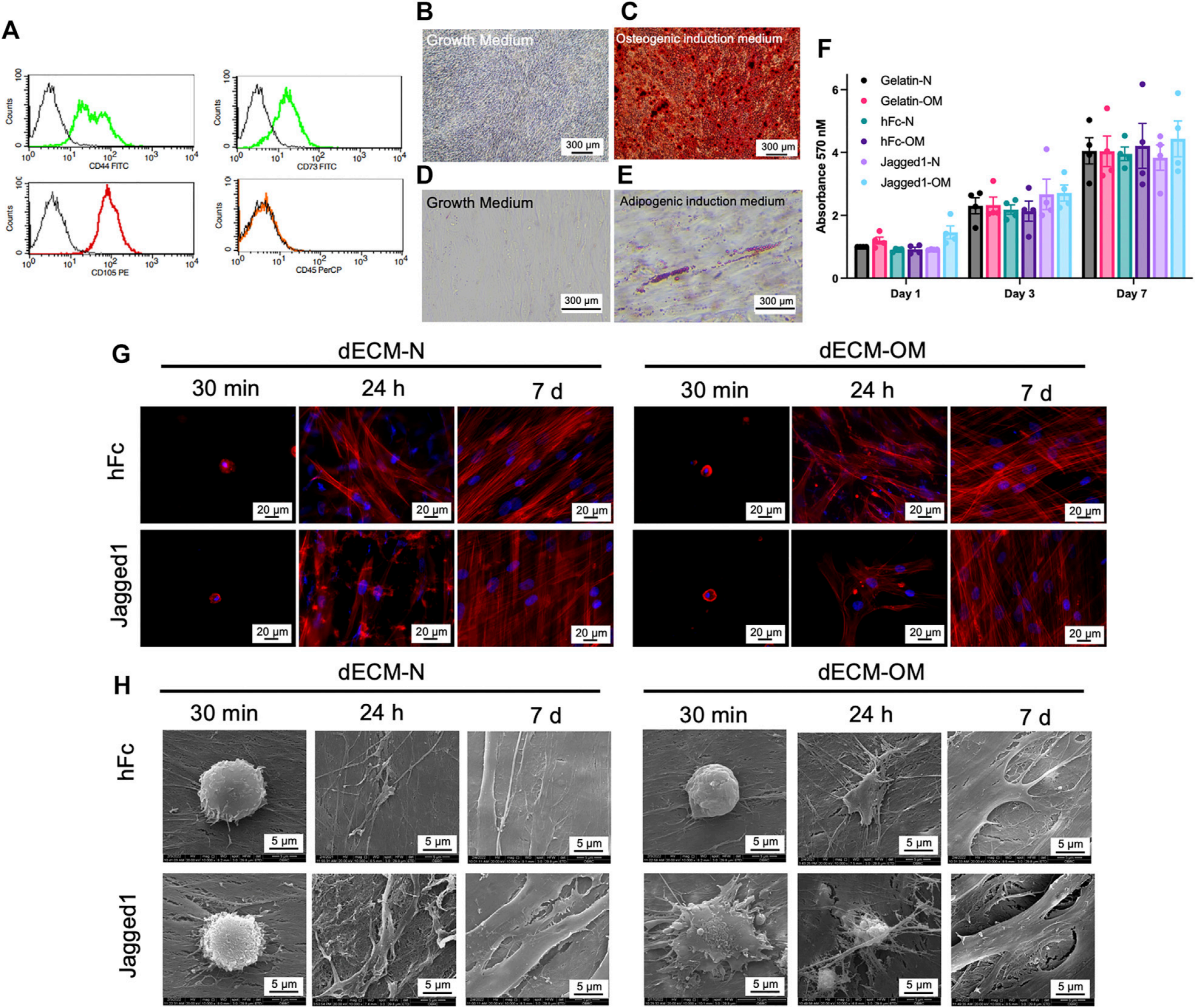
Biological responses of SCAPs on Jagged1 dECMs. Stem cells isolated from apical papilla (SCAPs) were characterized by flow cytometry to examine surface protein marker expression **(A)**. Mineralization was examined using Alizarin Red S staining on day 14 after osteogenic induction **(B–C)**. Intracellular lipid accumulation was detected using Oil Red O staining on day 16 after adipogenic induction **(D–E)**. Cell viability of SCAPs on dECM was determined using an MTT assay. The data were presented as mean ± SEM, and each dot represented the value from each donor **(F)**. Cell attachment and actin arrangement were examined using phalloidin staining at 30 min, 24 h, and 7 days **(G)**. Cell spreading was observed using scanning electron microscopic analysis **(H)**. dECM-N; decellularized extracellular matrix derived from maintaining cells in the normal medium, dECM-OM; decellularized extracellular matrix derived from maintaining cells in the osteogenic medium.

Stem cells isolated from apical papilla (SCAPs) were characterized by surface marker expression and multilineage differentiation ability. Isolated SCAPs expressed CD44, CD73, and CD105 but lacked CD45 expression (Figure 3A). Osteogenic and adipogenic differentiation of SCAPs was confirmed by the positive staining for mineral nodules by ARS staining (Figures 3B,C) and intracellular lipid droplets accumulation by Oil Red O staining (Figures 3D,E), respectively.

The biological responses of SCAPs on dECM derived from Jagged1-treated hDPSCs were next determined. SCAPs were reseeded on either Jagged1-or hFc-treated hDPSC-derived dECMs. Cell viability of SCAPs was assessed on days 1, 3, and 7 of culture by MTT assay. Gelatin-coated surfaces were used for the control. Even though gelatin is irreversibly denatured collagen, it still has a molecular structure and properties close to native collagen and has recently been widely utilized as a biomaterial scaffold (Tondera et al., 2016; Bello et al., 2020). Normal cell proliferation of SCAPs was observed in all control and tested surfaces (Figure 3F). Thus, Jagged1- and hFc-treated hDPSC-derived dECMs had no cytotoxicity and proliferative effects on SCAPs.

Cellular attachment and spreading were observed after seeding SCAPs on dECMs for 30 min, 24 h, and 7 days in the growth medium. The cytoskeletal protein organization, f-actin, was visualized by phalloidin immunofluorescence staining. SCAPs were attached to all dECM surfaces in 30 min without spreading. However, the well-organized f-actin arrangement in SCAPs was not noticeably different in either Jagged1-treated hDPSC-derived dECM-N or dECM-OM on day 7 (Figure 3G). SEM analysis further demonstrated that SCAPs attached, flattened, and spread with tiny filopodia on Jagged1-treated hDPSC-derived dECM-OM after 30 min, while on other surfaces the cell shape was still round (Figure 3H). At 24 h, SCAPs flattened and elongated on all dECM surfaces and later completely spread to form a monolayer covering the surface on day 7 (Figure 3H). These results suggested that all dECMs originated from Jagged1- and hFc-treated surfaces and are biocompatible for SCAPs proliferation, attachment, and spreading *in vitro*.

### dECM derived from Jagged1-treated hDPSCs promote mineralization of SCAPs

**FIGURE 4:**
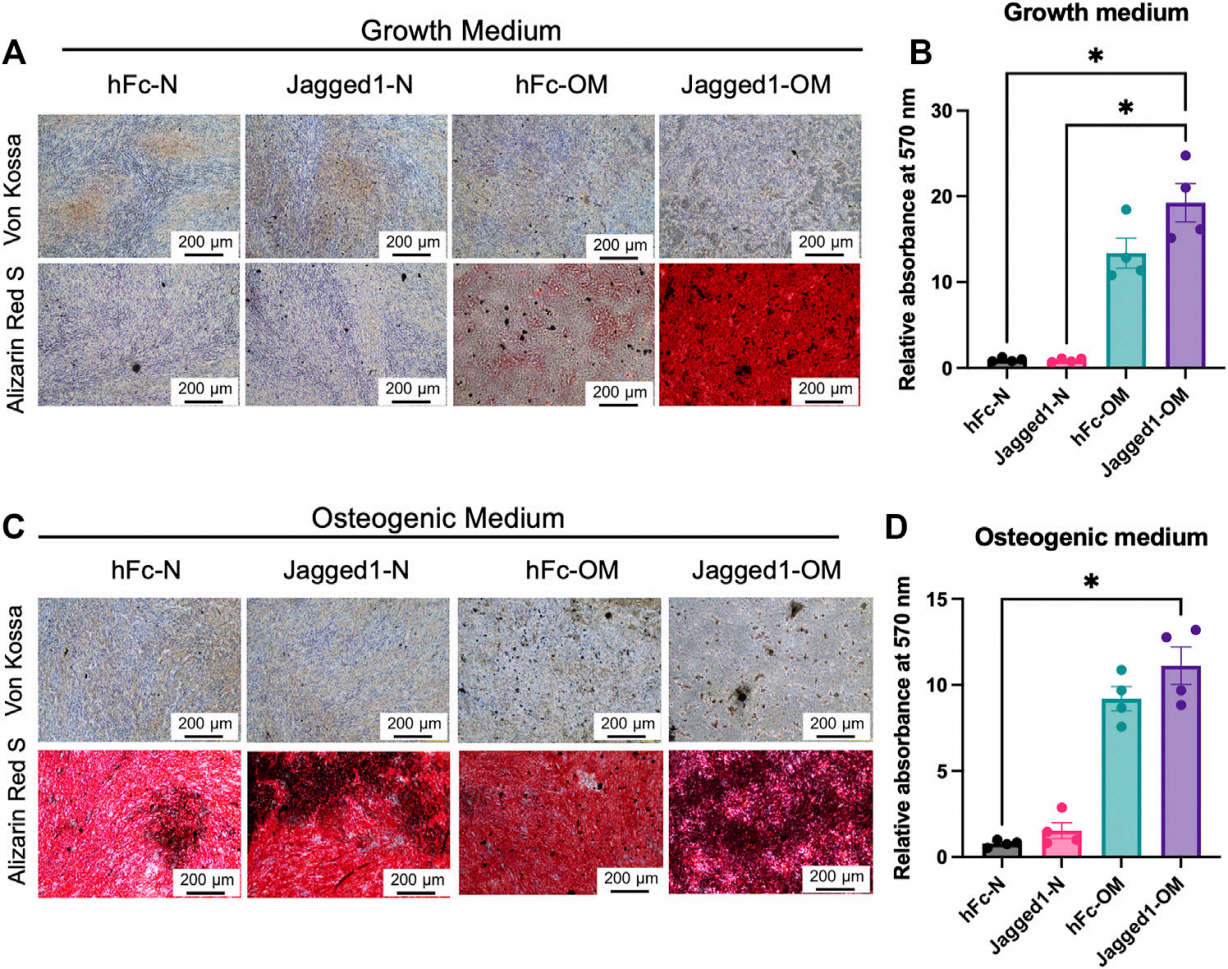
Jagged1-treated hDPSC dECM promotes mineralization ability of SCAPs. SCAPs were seeded on dECM and maintained in the growth medium **(A)** or osteogenic induction medium **(C)**. Mineralization was observed using Von Kossa and Alizarin Red S staining. The deposited Alizarin Red S was solubilized and the relative absorbance at 570 nm was illustrated **(B,D)**. Bars indicated a statistically significant difference.

To evaluate the osteogenic differentiation potential of SCAPs on Jagged1-treated hDPSC-derived dECMs from the normal medium (dECM-N) or OM (dECM-OM) conditions, mineralization was observed by ARS staining on day 7. SCAPs culturing on Jagged1-treated hDPSC-derived dECM-OM in the growth medium significantly increased calcium deposition compared with those on hFc-treated and Jagged1-treated hDPSC-derived dECM-N (Figures 4A,B). When SCAPs were maintained with OM for 7 days, those cells exhibited robustly increased *in vitro* mineralization. Cells on Jagged1-treated hDPSC-derived dECM-OM exhibited significantly higher mineralization compared with those cells on hFc-treated hDPSC-derived dECM-N (Figures 4C,D).

## Discussion

The ECM scaffold is a biomaterial candidate for prospective clinical application in tissue regeneration, repair, and remodeling of both skeletal and non-skeletal tissues (Alaribe et al., 2016; Lin et al., 2020). Decellularized brain tissue (bECM) rapidly augmented the development of mature neuronal networks (Lam et al., 2019). The adipose tissue-derived decellularized ECM also provided favorable graft properties for adipose tissue engineering when combined with adipose-derived stem cells to repair the soft tissue defects (Wang et al., 2013). Additionally, the ECM scaffold is an excellent source of bioactive proteins for mineralized tissue formation, including bone morphogenetic protein 2 (BMP2), transforming growth factor-beta (TGF β ), platelet-derived growth factor (PDGF), and vascular endothelial growth factor (VEGF) (Sonoyama et al., 2006; Ravindran et al., 2014a). Promotion of cell survival and proliferation after recellularized ECM scaffolds by increased telomerase activity was previously indicated (Sonoyama et al., 2006).

Activation of the canonical Notch signaling pathway by Jagged1 promotes the odonto/osteogenic differentiation of various dental stem cells. Indirect Jagged1 immobilized surfaces markedly enhanced the osteogenic marker genes expression such as *RUNX2, OSX, OCN, COL1, OPN, BMP2*, and *DSPP* in hDPCs, hPDLSCs, and human bone-derived cells (Osathanon et al., 2013; Manokawinchoke et al., 2017; Osathanon et al., 2019). Importantly, the differential gene expression profile of hDPSCs cultured on Jagged1 immobilized surfaces demonstrated the upregulation of several genes involved in ECM organization, focal adhesion, and ECM–receptor interaction (Manokawinchoke et al., 2017). Upregulation of some matrix metalloproteinase genes, *ADAMTS2, ADMATS3*, and *ADAMTS14,* which play a role in ECM proteins degradation and ECM remodeling, also supported the osteogenic differentiation and mineralization of hDPSCs. Previous studies revealed the pivotal functions of MMPs in osteogenic, chondrogenic, adipogenic, and endothelial differentiation (Mannello et al., 2006), mineral deposition, and calcium nodule during the differentiation process of MSCs (Almalki and Agrawal 2016). Moreover, ECM originating from hDPSCs served as an excellent material for inducing odontogenic differentiation of stem cells from the oral tissues (Ravindran et al., 2014b). Thus, hDPSC-derived ECM could be a promising biomaterial for utilization in regenerative dentistry, such as tooth and bone repair.

In this study, we generated and characterized the dECMs from immobilized Jagged1-treated hDPSCs and subsequently illustrated their osteoinductive activity on SCAPs. Previous reports showed that human gingival stem cells (hGSCs) could well attach, spread, and proliferate on hDPSC-derived dECMs (Nowwarote et al., 2021). In addition, hDPSC-derived ECM markedly enhanced the osteogenic differentiation of hGSCs (Nowwarote et al., 2021). Decellularization eliminates the genetic components while preserving proteins and ECM properties by chemical, enzymatic, physical, or combinative methods (Gilpin and Yang 2017). This procedure should be designed to trigger neither cytotoxicity nor immune response (Rieder et al., 2004). Thus, mechanical properties and reduced immunogenicity are critical considerations for further clinical applications of decellularized scaffolds (Gilpin and Yang 2017). dECMs in this study are biocompatible for recellularized SCAPs. Moreover, the criteria for evaluating appropriate decellularization included no visible nuclear material by staining with DAPI, less than 50 ng of dsDNA per mg of dry weight, and less than 200 bp of the DNA fragment (Gilbert et al., 2006). In addition, the protein components remaining in ECM after decellularization, such as collagen, fibronectin, and glycosaminoglycans (Crapo et al., 2011; Guneta et al., 2017), should be assessed. In accordance with those requirements, dECM-N and dECM-OM derived from Jagged1-treated hDPSCs were negative for DAPI staining and contained the classical ECM structure proteins, type I-collagen, and fibronectin, as well as those of the hFc-treated controls. Proteoglycans, which are composed of GAGs, are an abundant glycoprotein found in the ECM. It is important for the biological functions of ECM and is involved in ECM interaction with extracellular ligands for signaling transduction as well as stem cell homeostasis (Kular et al., 2014; Kusindarta and Wihadmadyatami 2018). We observed that hDPSC-derived dECMs treated on Jagged1 immobilized surfaces showed increased GAGs deposition, but there was no difference between normal and OM conditions. This suggested that the Jagged1-activated Notch signaling pathway participated in several gene expression and translational processes of ECM proteins. The potentiality of proteoglycans and GAGs in facilitating biological processes of stem cells was previously reported, which is a promising tool for developing as a bioactive material in bone regeneration (Chen et al., 2021).

Characterization of dental pulp stem cells in the *Jagged1-*knockout mouse model is not achievable due to embryonic lethality (Xue et al., 1999). However, we alternatively blocked Notch signaling pathway or knocked down the *NOTCH2* receptor by *γ*-secretase inhibitor or short hairpin RNA (shRNA), respectively. In both molecular strategies, Jagged1-induced osteogenic marker genes and mineralization were significantly eliminated in hDPSCs (Manokawinchoke et al., 2017).

The interactions between cells and ECM influenced cell responses and function. Because cell adhesion is important for cell communication and regulation, as well as tissue development and maintenance (Khalili and Ahmad 2015), biological scaffolds should encourage cell adhesion to the substrate that further modulates cell behaviors such as cell proliferation, migration, spreading, and differentiation. We showed that Jagged1-treated dECM-OM promoted early cell adhesion and spreading of SCAPs compared to other dECMs by 30 min. It has been reported that cell shape and spreading area are related to the osteogenic differentiation potential (Jiao et al., 2020). Early cell adhesion and spreading correspond to the increase of osteogenic differentiation of human mesenchymal stromal cells on bone substituted materials (Barradas et al., 2013). Hence, the early spreading of SCAPs on Jagged1-treated hDPSC-derived dECM-OM could be related to its higher mineralization when cultured in an osteogenic induction medium.

SCAPs are the neural crest-derived cells located underneath dental pulp tissues at the apex of the developing roots (Sonoyama et al., 2008). Since the dental papilla contributes to dentin and dental pulp tissue formation, the potential of SCAPs for tooth repair or endodontic regeneration should be expected. Regenerative endodontic treatment is involved in the regeneration of the pulp–dentin complex (Raddall et al., 2019). In this therapy, the advantages of biomaterials scaffold were used to improve the treatment outcome. The scaffold was used to transplant into the root canal, which led to the migration of SCAPs into the scaffold to induce revascularization and root development (Raddall et al., 2019; Widbiller and Schmalz 2021). The previous studies demonstrated that SCAP-mediated tissue regeneration suggests promising cell-based therapy for root regeneration. *In vivo*, when SCAPs were added into a root-shaped HA/TCP block with PDLSCs, they supported the restoration of tooth function in swine (Sonoyama et al., 2006)**.** The previous studies also reported that Jagged1 is essential for odonto/osteogenic differentiation and osteoblast development (Hill et al., 2014; Manokawinchoke et al., 2017). Dental pulp-derived ECM provided a proper environmental niche to assist cell replication and regeneration of dental pulp tissues (Zhang et al., 2017). Decellularized dental pulp scaffold supported the proliferation and differentiation of odontoblast-like cells of SCAPs (Song et al., 2017). Therefore, we hypothesized that Jagged1-derived dECM could have the capability to enhance odontogenesis/osteogenesis of dental stem cells including SCAPs. Interestingly, we found the higher mineralization of SCAPs after reseeding on Jagged1-OM dECMs by ARS staining in both the normal medium and OM. The hFc-treated hDPSCs dECM did not show the remaining mineral content. This observation was consistent with a previous study showing that decellularized process could eliminate the mineral content in dECM of the hDPSCs culture in the OM medium (Nowwarote et al., 2021). However, in the present study, we noted that decellularization could not eliminate mineral components in Jagged-1 treated hDPSCs dECM. This observation could imply the distinct characteristics of ECM in Jagged1-treated hDPSCs as the mineral crystals could tightly bind to the ECM structure. An additional investigation should be employed to elucidate the underlying mechanism.

Further studies are needed to specify the properties of Jagged1-treated dECM and the mechanism to augment the mineralization of SCAPs and other dental stem cells, for example, the role of ECM proteins. Indeed, the development of cell-derived ECM scaffold structures with other materials to stabilize the mechanical and physical properties as well as improve the recellularization and regeneration are also required. In summary, Jagged1-treated hDPSCs dECM could be beneficial to develop as the novel allograft for alternative dental therapeutics such as regenerative endodontic procedures or pulp capping material.

## Conclusion

In conclusion, dECMs derived from indirect immobilized Jagged1-treated hDPSCs increase *in vitro* odonto/osteogenic differentiation and have no toxic effect on SCAPs, which suggests the potential of Jagged1 dECMs for further applications in regenerative dentistry.

## Data Availability

The datasets presented in this study can be found in online repositories. The names of the repository/repositories and accession number(s) can be found in the article/Supplementary Material.
